# Polystyrene-based Hollow Microsphere Synthesized by *γ*-ray Irradiation-assisted Polymerization and Self-Assembly and Its Application in Detection of Ionizing Radiation

**DOI:** 10.1038/srep41876

**Published:** 2017-01-31

**Authors:** Wenhui Fan, Qing Li, Liang Hu, Siqi Yan, Wanxin Wen, Zhifang Chai, Hanzhou Liu

**Affiliations:** 1 School of Radiation Medicine and Protection, and School for Radiological and Interdisciplinary Sciences (RAD-X), Medical College of Soochow University, Suzhou, Jiangsu 215123, P.R. China

## Abstract

To simply and multitudinously synthesize hollow microspheres in a pure system is important for relevant research and application. Here, a simple and novel one-pot synthetic strategy to prepare polystyrene (PS) hollow microspheres *via* irradiation-assisted free-radical polymerizing and self-assembly (IFPS) approach under *γ*-ray irradiation with no additives introduced into the system is presented. And PS/2,5-Diphenyloxazole (PPO) fluorescent microspheres have been prepared successfully by IFPS reaction, which can be used as scintillators for the detection of ionizing radiation. A linear relationship between emitted luminescence and dose-activity in water is obtained, which suggests that composite microspheres could be used as liquid scintillation in specific environment.

Hollow microspheres with unique physical and chemical properties have attracted great attentions because of their wide applications in drug delivery, energy storage conversion, and micro-reactors, *etc*.^1^,^2^,^3^,^4^,^5^. Polystyrene (PS) and its derivatives with high steric hindrance and chemical durability have been used widely for designing and fabricating hollow microspheres. Zhang *et al*. reported double-shell hollow microspheres containing polystyrene prepared by macromolecular self-assembly process^6^, Moreover, Deng and Sun synthesized temperature-sensitive hollow microspheres by using divinylbenzene as the crosslinking agent^7^. Block copolymers with polystyrene have been also applied to preparing hollow microspheres^8^. However, there are few studies on the preparation of uniform PS hollow microspheres, which mainly focused on complicated double emulsions polymerization method^9^.

Therefore, it is still a challenge to produce hollow microspheres in large scale with few additives, which is indispensable for its wide application. Template^10^,^11^, emulsion polymerization^12^ and self-assembly^13^,^14^,^15^ are the most common methods to prepare organic hollow microspheres, with which polymeric hollow microspheres of various structures and forms are prepared and reported in recent years^16^,^17^. These approaches usually require specific templates, emulsifiers and chemical groups, as well as unique preparation progress, which cannot satisfy the requirements of mass production. Therefore, it is of scientific and practical significance to set up a novel, simple and practicable route for synthesizing hollow microspheres.

Gama (*γ*)-irradiation induced polymerization and crosslinking is a simple, pure and effective method extensively used to synthesize polymer nano- or micro-materials^18^,^19^. By *γ*-irradiation in micro-emulsion and Pickering emulsions, polymer hollow spheres can be prepared^20^,^21^. In addition, organic- and inorganic- polymer composites hollow spheres can also be obtained by *γ*-irradiation^22^,^23^. In the preparation of hollow microspheres by irradiation, emulsifier and stabilizer play a crucial role. There is no published report so far to prepare polymeric hollow microspheres without emulsifier, stabilizer, initiator, auxiliary reagent.

In this paper, PS hollow microspheres are synthesized under *γ*-ray irradiation at room temperature *via* irradiation-assisted free-radical polymerizing and self-assembly (IFPS) reaction, in which no additive is introduced. A possible growth mechanism of IFPS for the preparation of hollow spheres is also presented. Moreover, PS/PPO composite microspheres are prepared by IFPS, which can be used as scintillators for the detection of ionizing radiation in aqueous and non-aqueous environment.

## Results

### PS hollow microspheres

**Figure 1:**
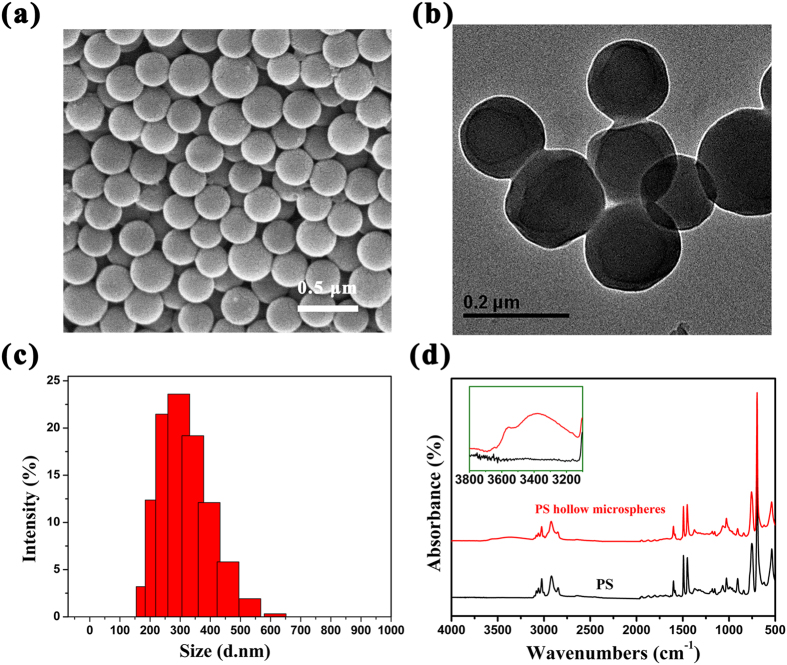
The structural properties of PS hollow microspheres measured by (**a**) SEM; (**b**) TEM; (**c**) Diameter distribution and zeta potential; (**d**) FT-IR spectroscopy. The ratio of styrene to deionized water was 5% in reaction system.

PS hollow microspheres are prepared by a simple and gentle one-pot IFPS method. As shown in Fig. 1(a), the diameter of obtained PS spheres is of 300 nm approximately. The spheres are found to be hollow in Fig. 1(b) and Figure S1, moreover, the distinct pale center and dark edge can be observed clearly^24^. The transmission electron microscopy (TEM) images of broken microspheres by ultrasound are shown in Figure S2, in which the hollow microspheres structure can be observed clearly. In addition, these hollow spheres exhibit a diameter in the range of 200–500 nm and an averages diameter around 300 nm (Fig. 1(c)), which indicates that uniform PS hollow microspheres can be prepared by IFPS method.

**Figure 2:**
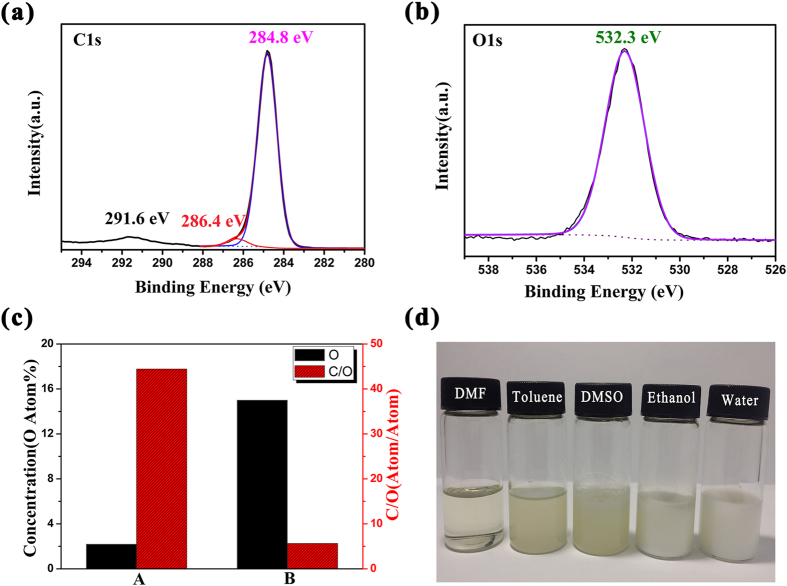
(**a**) C1s and (**b**) O1s spectra of XPS study; (**c**) Oxygen content and C/O (atom/atom) of hollow microspheres by using XPS spectroscopy and SEM-EDS element analysis; (**d**) The dispersibility of PS hollow microspheres in water, ethanol, DMSO, DMF and Toluene solution, respectively. The reaction condition was the same as Fig. 1.

For comparison, PS polymer is also synthesized with the same approach in the absence of deionized water. The chemical structures of PS hollow microspheres and PS polymer are as shown in Fig. 1(d), in which the obtained PS hollow microspheres feature the C-H stretching of benzene ring at 3000–3105 cm^−1^ and the C-H stretching vibration of alkyl groups at 2930 and 2850 cm^−1^, respectively^25^. Similarly, these peaks can be easily observed in the spectrum of PS polymer, which suggests that PS hollow microspheres are successfully obtained. In addition, it is remarkable that a new absorption band also appears at 3,300 cm^−1^ in the spectrum of the PS hollow microspheres that can be attributed to a hydroxyl groups which can also be determined by XPS study (Fig. 2(a,b)). The spectrum of hollow microspheres in Fig. 2(a) can be deconvoluted into three different peaks that correspond to carbon atoms with the assumption of different binding statues, while one peak appears in the spectrum of O1s (Fig. 2(b)). The peaks centering at the binding energies of 291.6, 286.4 and 284.8 eV are assigned to the carbon atoms of C=C in benzene ring, C-OH (hydroxyl) and C-C in polystyrene, respectively, and O1s peak at 532.3 eV is assigned to the oxygen atoms of hydroxyl groups. Additionally, oxygen can also be proved to exist in microspheres by XPS spectroscopy and SEM-EDS element analysis, and its contents are 15.01atom% and 2.20atom %, respectively (Fig. 2(c)), indicating that more oxygen or oxidation groups are dispersed on the surface of microspheres due to different detection depth of test methods. To sump the points elaborated above, PS hollow microspheres with many hydroxyl groups on their surface are prepared successfully by *γ*-ray irradiation.

Pristine PS microspheres are not able to disperse in water due to the hydrophobicity, which limits their applications severally. However, it can be overcome by the introduction of some hydrophilic groups into PS chains effectively^26^. In this paper, the PS hollow microspheres prepared exhibit remarkable dispersion in ethanol and dimethyl sulphoxide (DMSO) (Fig. 2(d)) within 24 h, and water, toluene and N,N-Dimethyl formamide (DMF) longer than 1 month, due to the existence of hydroxyl groups.

### Irradiation-assisted free-radical polymerizing and self-assembly reaction

**Figure 3:**
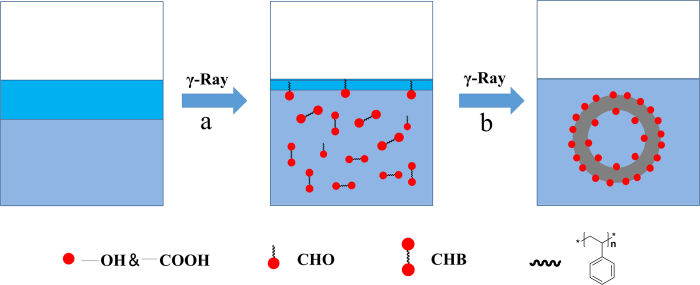
The mechanism of IFPS. (**a**) Irradiation free radical addition and polymerization process; (**b**) Irradiation-induced self-assembly and polymerization process.

**Figure 4:**
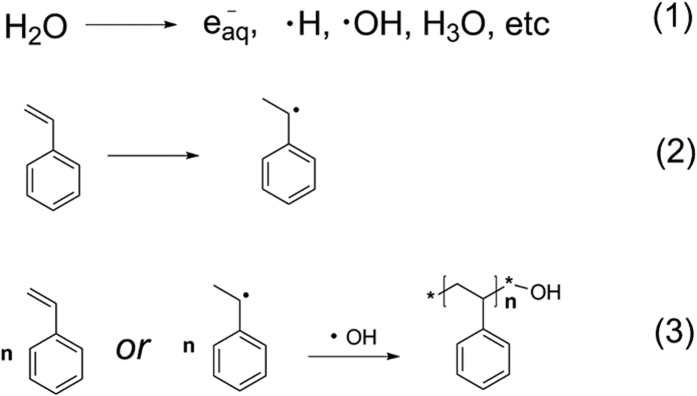
Chemical reaction under *γ*-ray irradiation in the reaction system.

The formation of microspheres might be divided into two stages as shown in Fig. 3 a, Irradiation free radical addition and polymerization. Polymer chain with hydroxyl groups will be acquired *via* monomer free radical polymerization and free radical addition reaction with hydroxyl radicals as radical initiator and terminator. The possible reactions in system can be seen in Fig. 4 b, Irradiation-induced self-assembly and polymerization process. There are two kinds of PS chains in water, chain segment with hydroxyl group at one end (CHO) and chain with hydroxyl groups at both ends (CHB). The hollow emulsion with PS chain, seeming the same as “water-in-oil-in water” (W/O/W) type double emulsion^27^, is formed by self-assembly with CHO/CHB as an emulsifying agent. And polymerization reaction occurs at the same time in the process of self-assembly to form the stable hollow microspheres structure. The shape of hollow is determined by the proportions of different chain segments and absorbed dose. The two stages are carried out simultaneously under continuous irradiation. This method is named as “Irradiation-assisted Free-radical Polymerizing and Self-assembly (IFPS)” which could be used to prepare hollow microspheres with hydrophobic monomers and water solution under irradiation.

**Figure 5:**
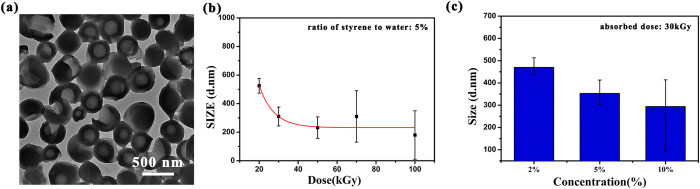
PS hollow microspheres prepared by different reaction condition. (**a**) TEM analysis of PS hollow microspheres prepared in the presence of oxygen; (**b**) The size of microspheres with different absorbed dose and (**c**) different concentration ratio of monomer to water. The reaction condition was the same as Fig. 1.

Additionally, the oxygen in the reaction would hinder the polymerization due to its free radical eliminating action, which would lead to broken and irregular PS fragments and microspheres in water (Fig. 5(a)). The size, shape and uniformity of PS microspheres are significantly affected by concentration ratio of monomer to water and absorbed dose. Larger-size microspheres could be prepared at lower absorbed dose or higher concentration ratio of styrene to water, and the size dimension of PS microspheres become more irregular with the increasing absorbed dose (Fig. 5(b,c)), which can also be found in the SEM analysis of microspheres under different reaction conditions (Figure S3). And the zeta potential decreases with increasing absorbed dose, implying that the hollow microspheres become instable at higher absorbed dose (Figure S4), which is in good agreement with the experimental results of size and SEM analysis. The conversion of styrene gel fraction of resultant polystyrene hollow microspheres was exceeded 80% with 2% and 5% concentration under 20–50 kGy absorbed dose, while its was less than 60% with 10% concentration due to the limitation of reaction interface (Figure S5).

### PS/PPO fluorescent microspheres

**Figure 6:**
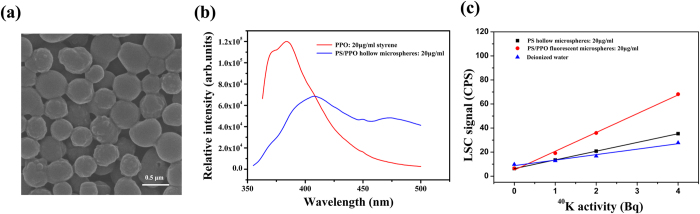
(**a**) The SEM analysis of raspberry-shape morphology for PS/PPO microspheres. (**b**) The emission spectrum of PPO and PS/PPO microspheres. (**c**) The LSC signal of ^40^K (14.4 Bq/g) measured by liquid scntillation counter with PS hollow microspheres, PS/PPO microspheres and water solution, where the measured time was 5 minutes.

It is of great significance to change materials’ hydrophobicity/hydrophilicity or dispersibility in different solvents, such as liquid chemical method, grafting technology, and blending technology. PPO is a common organic scintillator, whose dimethylbenzene solution has been extensively used in liquid scintillation method^28^. However, PPO can not be dissolved and dispersed in the water, which limits its application in detection of ionizing radiation, especially tritiated water (HTO) and ^40^K, *etc*., Besides, the emission wavelength of liquid scintillator (PPO in pure solvent) appeares in 368 nm and 383 nm, which is disadvantageous to optical signal acquisition of photomultiplier, and thus a second scintillator is required for increasing emission wavelength^29^,^30^. In this paper, PPO are loaded to the PS hollow microspheres by IFPS one-step method, which shows good hydrophobicity/hydrophilicity and ionizing radiation detection capability. The structure and morphology of prepared PS/PPO fluorescent microspheres are as shown in Fig. 6. They are of raspberry-shape, and the smaller spherical protrusions on the surface are PPO. (Fig. 6(a)). XPS analysis and SEM-EDS element analysis show that the microspheres are composed of PPO and PS (Figures S6 and S7), with the emission wavelength of composite microspheres appeared in 408 nm and 474 nm (Fig. 6(b)), which is beneficial for liquid scintillation detection.

PS/PPO microspheres water solution is emulsion not pellucid solution, which means optical yield of solution is affected. Tritium water is used to determine the optimal concentration, and the LSC signal generated by microspheres concentrations ranging from 0 to 20 g/L at tritium water with defined activity is measured. The results show that 5 g/L microspheres with 5.7 mmol/L PPO provides an adequate signal (Figure S8). The radiation emission behavior of PS/PPO fluorescent microspheres, PS hollow microspheres and water are counted on LCS by measuring the defined activity of ^40^K. The ^40^K was standard sources that can be used to precisely measure the radiation emission behavior of radionuclide, while the tritium water was configured in lab. As shown in Fig. 6(c), there is a linear relationship (*r*^2^ = 0.99998) between LSC signal and the dose ranging from 1 Bq to 7 Bq, indicating that the PS/PPO fluorescent microspheres can be used as scintillator. Moreover, this linearity shows that the respond is constant even after being exposed to larger doses, which is hinted that fluorescent microspheres could exhibit a high longevity with respect to the absorbed dose at aqueous solution^31^. In addition, the PS/PPO fluorescent microspheres have high detection efficiency in DMF/Toluene solution to *γ*-ray irradiation (Figure S9), particularly in Toluene solution, showing their excellent performance in detecting ionizing radiation for organic solvents.

## Conclusions

A simple and novel one-pot synthetic strategy to prepare PS hollow microspheres *via* IFPS approach under *γ*-ray irradiation has been demonstrated. The results show that different PS-based hollow microspheres with a controllable size have a real empty core and a polymer shell structure. The hollow-structure microspheres can be dispersed in water excellently due to the existence of hydroxyl groups on the surface. In addition, the reaction occurs in maximum purity system without additives, which ensures the purity of samples. The growth mechanism and methodology exhibit that the IFPS reaction has potential for producing hollow microspheres in large-scale at low cost. And it is an emulsion polymerization and self-assembly process, which shows the possibility of more components to be introduced and more structures to be synthesized. The PS/PPO microspheres prepared with IFPS reaction is proved to have excellent ionizing radiation detection capability in different solvents, which suggest that PS/PPO fluorescent microspheres could be used as liquid scintillation in specific environment.

## Methods

### Chemicals

Styrene (>99.0%), Ethanol (>99.9%), toluene (>99.0%), acetone (>99.0%), DMF (>99.9%) and DMSO (>99.9%) were purchased from Sinopharm Chemical Reagent Co. Ltd. PPO was purchased from BBI life sciences. ^40^K and ^3^H came from National Institute of Metrology. Deionized water was purified by Direct Q5 pure water system.

### PS hollow microspheres

The PS hollow microspheres were simply prepared by styrene/deionized water mixed solution under ^60^Co *γ*-ray irradiation. The styrene and deionized water with different ratio were put into irradiation glass tube, and the tube was purged with nitrogen for 20 minutes to remove oxygen and sealed. And then the samples were irradiated with ^60^Co *γ*-ray source at a constant dose rate of 2 kGy.h^−1^ for 30 kGy absorbed dose at room temperature. After the reaction, the samples were purified by centrifuge at 10000 rpm for 10 min to get uniform polystyrene microspheres. Finally, the samples were vacuum freeze-dried to constant weight. The sample had been prepared by the above method in the presence of oxygen.

PS hollow microspheres was visualized using Scanning Electron Microscopy (SEM, S-4700 FE-Scanning electron microscopy, Japan) and transmission electron microscopy (TEM, Tecnai G2 spirit BioTwin, USA). The hollow microspheres were stable dispersed in deionized water, and their size and zeta potential were measured by Nano particle and zeta potential analyzer (Zetasizer Nano ZS90, UK). In addition, the dispersibility of microspheres was measured by dispersing 5% (m/m) hollow microspheres in different solvents (water, ethanol, DMF, DMSO, and Toluene solution).

The chemical structure of PS hollow microspheres was analyzed by fourier transform infrared spectroscopy (FI-IR, Thermo iS50, USA). C1s and O1s spectra of PS microspheres were investigated by X-ray photoelectron Spectroscopy (XPS, ESCALAB 250Xi, USA). In addition, Oxygen content and C/O (atom/atom) of hollow microspheres could be analyzed by XPS spectroscopy and energy dispersive spectrometry (EDS).

### Pristine polystyrene

Styrene was put into special irradiation tubes, and then purged with nitrogen for 20 min and sealed, irradiated by ^60^Co *γ*-ray source at a constant dose rate of 2 kGy.h^−1^ at room temperature for 30 kGy absorbed dose. The sample was dried by vacuum freeze-dying.

### PS/PPO fluorescent microspheres

2,5-diphenyl oxazole (PPO 0.02 g) powder was dissolved in styrene (2 ml), and mixed with deionized water (98 ml) in the special irradiation tubes, then irradiated by ^60^Co *γ*-ray at a constant dose rate of 2 kGy.h^−1^ at room temperature for 30 kGy to form fluorescent microspheres. Vacuum freeze-dying was adapted to dry samples.

The morphology of PS/PPO fluorescent microspheres was measured by SEM. The excitation and emission spectrum of PS/PPO fluorescent microspheres were conducted by steady transient fluorescence spectrometer (STFS). To observe the radiation sensitivity of PS/PPO fluorescent microspheres, different activity of ^40^K (14.4 Bq/g) was added in solution, and then the LSC signal was obtained by X-ray irradiation apparatus (RS-2000 Pro, USA).

## Additional Information

**How to cite this article**: Fan, W. *et al*. Polystyrene-based Hollow Microsphere Synthesized by *γ*-ray Irradiation-assisted Polymerization and Self-Assembly and Its Application in Detection of Ionizing Radiation. *Sci. Rep.* **7**, 41876; doi: 10.1038/srep41876 (2017).

**Publisher's note:** Springer Nature remains neutral with regard to jurisdictional claims in published maps and institutional affiliations.

## Supplementary Material

Supplementary Information
