# Supplementary material for: Polystyrene-based Hollow Microsphere Synthesized by γ-ray Irradiation-assisted Polymerization and Self-Assembly and Its Application in Detection of Ionizing Radiation
Source: Sci Rep. 2017 Jan 31;7:41876. doi: 10.1038/srep41876 (PMC5282558; doi:10.1038/srep41876)
Supplement: Supplementary Information [file srep41876-s1.pdf]

Supporting Information

Polystyrene-based Hollow Microsphere Synthesized by γ-ray Irradiation-assisted Polymerization and Self-Assembly and Its Application in Detection of Ionizing Radiation

Wenhui Fan, Qing Li, Liang Hu, Siqi Yan, Wanxin Wen, Zhifang Chai, Hanzhou Liu*

School of Radiation Medicine and Protection, and School for Radiological and Interdisciplinary Sciences (RAD-X), Medical College of Soochow University. Suzhou , Jiangsu 215123, P.R. China.

*Corresponding author.

E-mail address: hzhliu@suda.edu.cn

[+]These authors contributed equally to this work.

Supplemental Figures

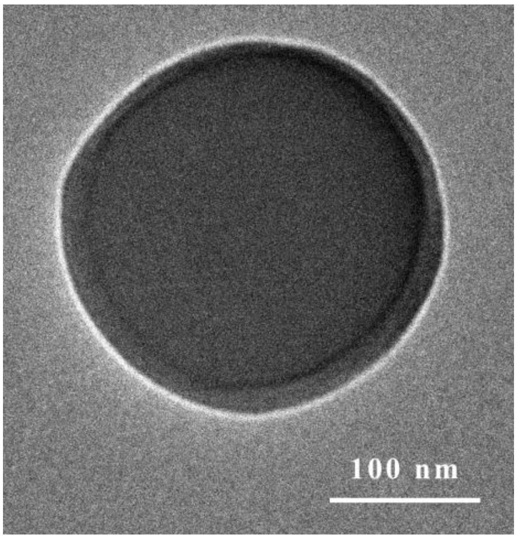

Figure S1 The hollow structure of PS microspheres measured by TEM analysis.

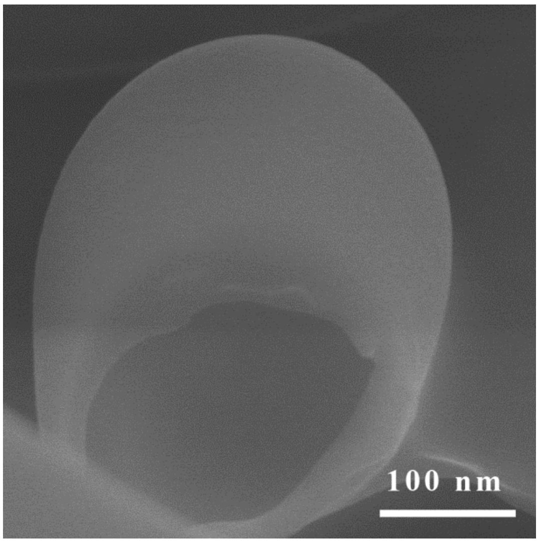

Figure S2 TEM images of broken PS microspheres by ultrasound 30mins.

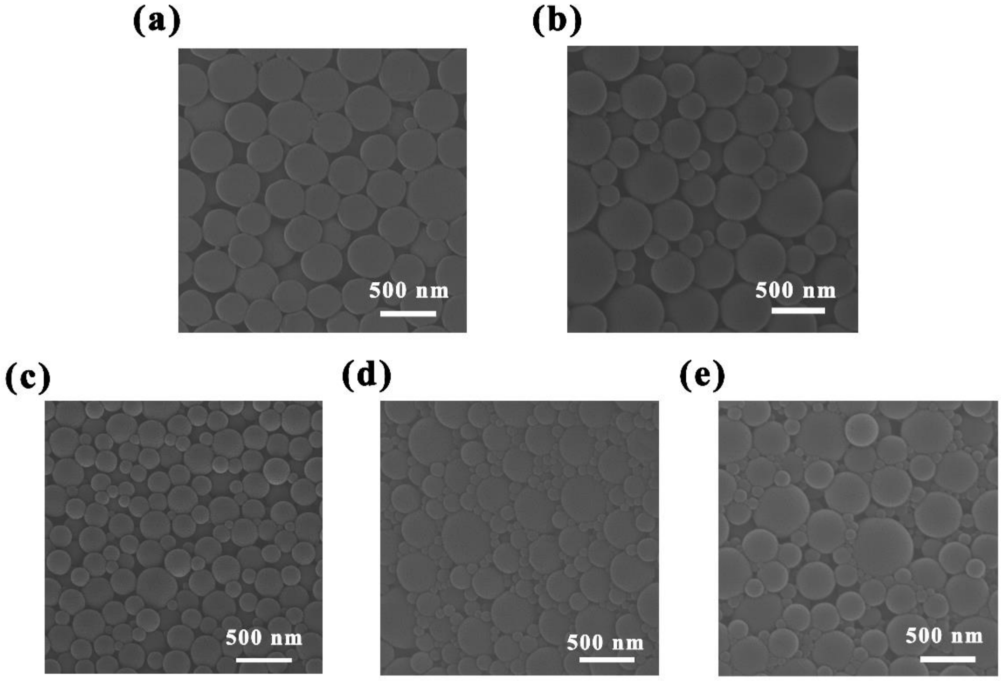

Figure S3. Different concentrations ratio of monomer to water and absorbed doses have multiply affected on hybrid hollow microspheres. With the increase of concentration ratio of monomer to water and absorbed dose, the size of hybrid hollow microspheres become smaller and irregular,(a) 2%; (b) 10%;(c)50kGy;(d) 70kGy;(e) 100kGy.

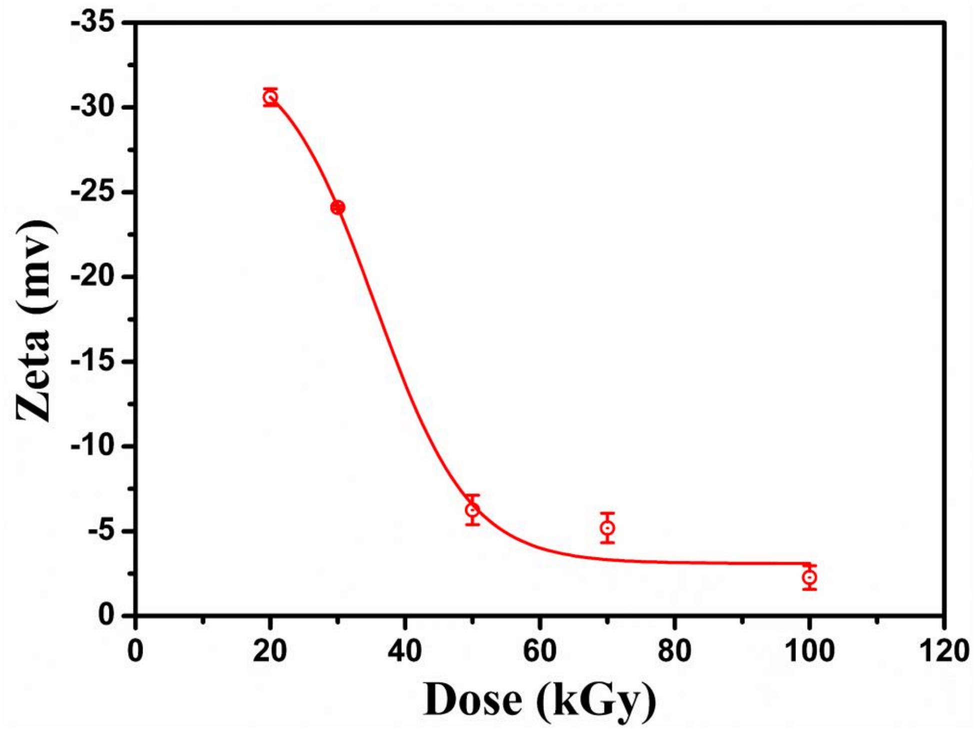

Figure S4. With the increase of absorbed doses, the zeta potential is decreasing at the same time.

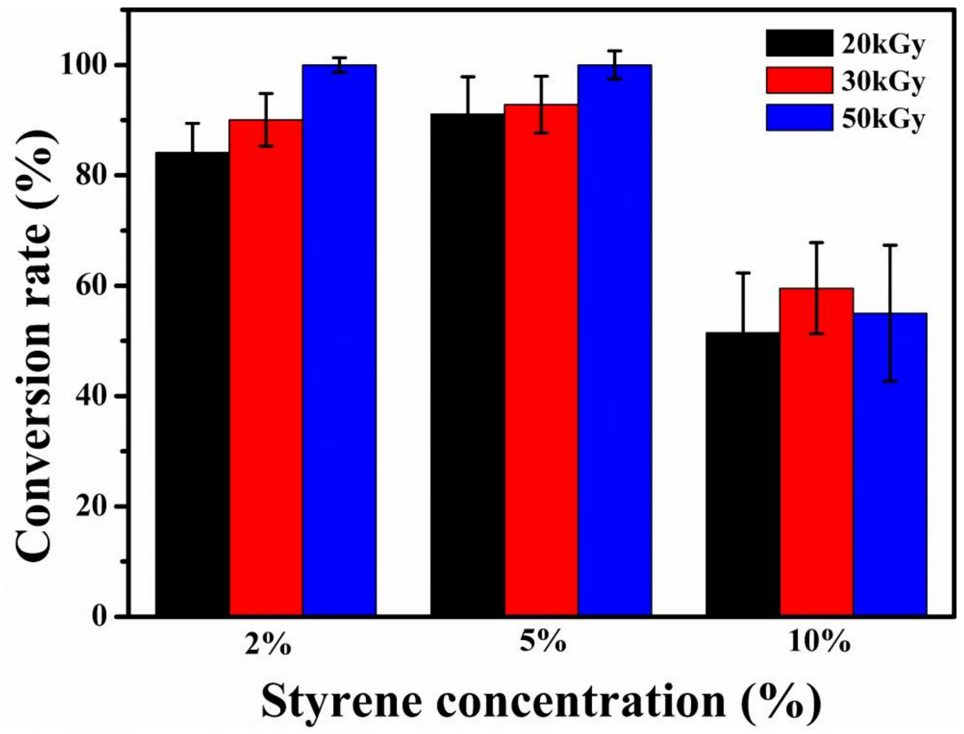

Figure S5. The conversion of styrene gel fraction of resultant polystyrene hollow microspheres with different reaction condition.

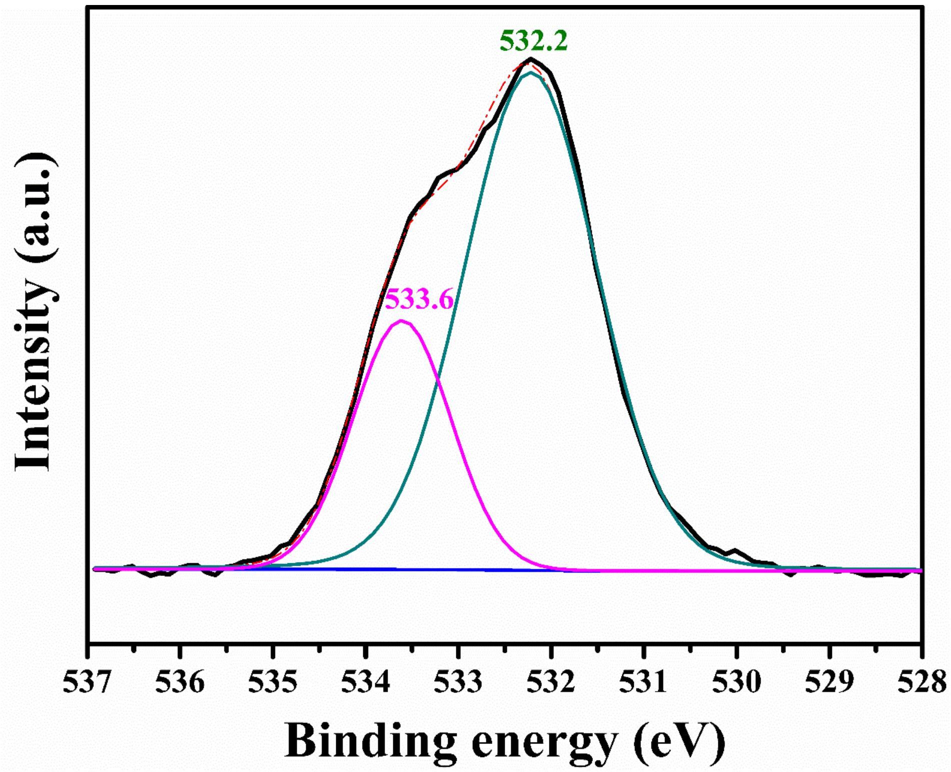

Figure S6. The O1s spectra of XPS study of PS/PPO microspheres. O1s peaks at 532.2eV and 533.6 were assigned to the oxygen atoms of hydroxyl groups of PS microspheres and ether groups of PPO, respectively.

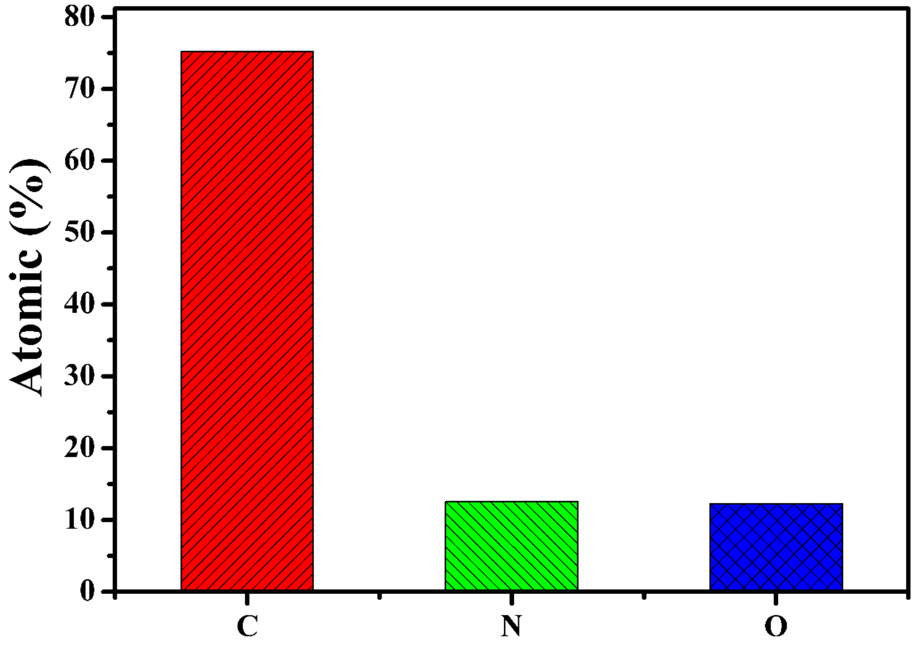

Figure S7. Oxygen content and C/O (atom/atom) of PS/PPO microspheres by SEM-EDS element analysis.

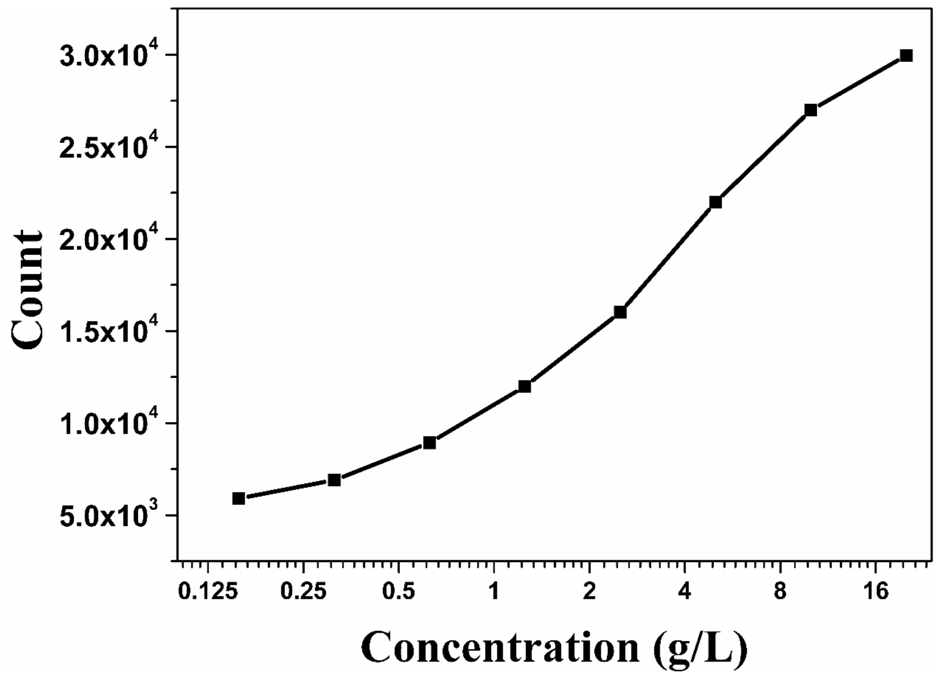

Figure S8. PS/PPO microspheres solution is not transparent, which means their concentration will greatly influence for optical yield. Using 63μCi/ml of ^{3}H 5μL, it was we measured the signal generated by PS/PPO microspheres concentrations from 0 to 20g/L for 5 minutes. It was determined experimentally that 5g/L provided an adequate signal.

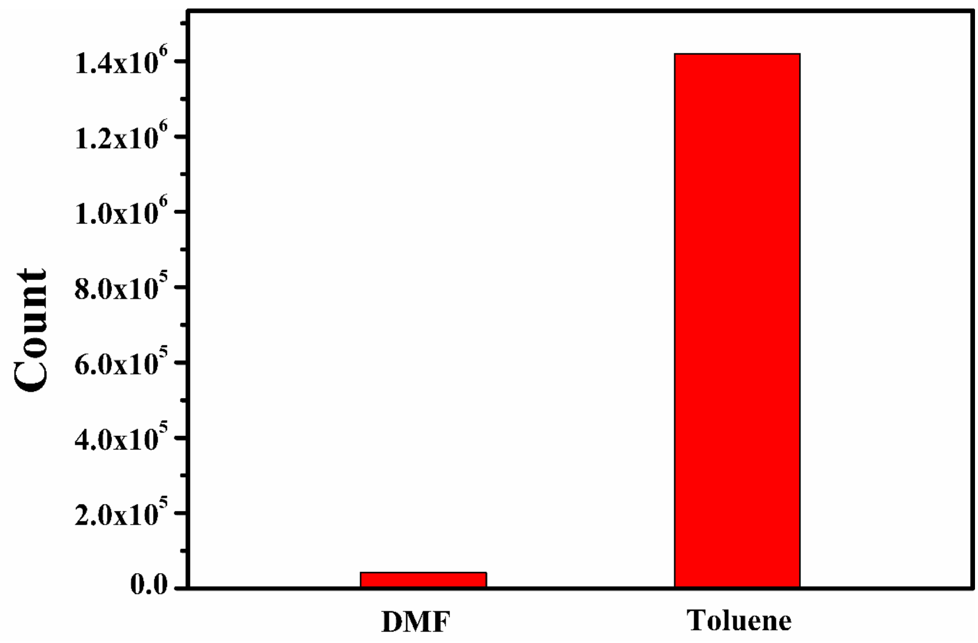

Figure S9. 5g/L PS/PPO microspheres solution has excellent detection efficiency at DMF/Toluene solution for *γ*-ray. It is measured count by using 63μCi/ml of ^{3}H 5μLfor 5 minutes.
